# Operationalising Factors That Explain the Emergence of Infectious Diseases: A Case Study of the Human Campylobacteriosis Epidemic

**DOI:** 10.1371/journal.pone.0079331

**Published:** 2013-11-21

**Authors:** Norval J. C. Strachan, Ovidiu Rotariu, Marion MacRae, Samuel K. Sheppard, Alison Smith-Palmer, John Cowden, Martin C. J. Maiden, Ken J. Forbes

**Affiliations:** 1 Institute of Biological and Environmental Sciences, University of Aberdeen, Aberdeen, United Kingdom; 2 School of Medicine and Dentistry, University of Aberdeen, Aberdeen, United Kingdom; 3 Medical Microbiology and Infectious Diseases, Swansea University, College of Medicine, Swansea, United Kingdom; 4 Health Protection Scotland, National Services Scotland, Glasgow, United Kingdom; 5 Department of Zoology, University of Oxford, Oxford, United Kingdom; National Institute for Viral Disease Control and Prevention, CDC, China

## Abstract

A framework of general factors for infectious disease emergence was made operational for *Campylobacter* utilising explanatory variables including time series and risk factor data. These variables were generated using a combination of empirical epidemiology, case-case and case-control studies, time series analysis, and microbial sub-typing (source attribution, diversity, genetic distance) to unravel the changing/emerging aetiology of human campylobacteriosis. The study focused on Scotland between 1990–2012 where there was a 75% increase in reported cases that included >300% increase in the elderly and 50% decrease in young children. During this period there were three phases 1990–2000 a 75% rise and a 20% fall to 2006, followed by a 19% resurgence. The rise coincided with expansions in the poultry industry, consumption of chicken, and a shift from rural to urban cases. The post-2000 fall occurred across all groups apart from the elderly and coincided with a drop of the prevalence of *Campylobacter* in chicken and a higher proportion of rural cases. The increase in the elderly was associated with uptake of proton pump inhibitors. During the resurgence the increase was predominantly in adults and the elderly, again there was increasing use of PPIs and high prevalences in chicken and ruminants. Cases associated with foreign travel during the study also increased from 9% to a peak of 16% in 2006 before falling to an estimated 10% in 2011, predominantly in adults and older children. During all three periods source attribution, genetic distance, and diversity measurements placed human isolates most similar to those in chickens. A combination of emergence factors generic for infectious diseases were responsible for the *Campylobacter* epidemic. It was possible to use these to obtain a putative explanation for the changes in human disease and the potential to make an informed view of how incidence rates may change in the future.

## Introduction

Emerging infectious diseases can be defined as “infections that have newly appeared in a population or have existed but are rapidly increasing in incidence or geographic range [1].” Most emerging infectious diseases are of zoonotic origin and consequently involve spill over from animal to human populations [2], [3]. A number of factors can contribute to the emergence of an infectious disease and these include (i) ecological changes (including those due to economic development and agricultural land use), (ii) human demographics, behaviour, (iii) international travel and commerce (iv) technology and industry (v) microbial adaptation/change and (vi) breakdown in public health measures [1].

*Campylobacter* is recognised as the largest cause of bacterial gastroenteritis in the developed world [4] with 70,973 cases reported in the UK during 2011 [5], [6], >220,000 in the EU [7] and an estimated 850,000 foodborne domestically acquired cases in the USA annually [8]. Since there is significant underreporting the actual number of community cases is likely to be considerably higher (e.g. estimated to be nine-fold higher in the UK [9]). Further, around 10% of indivduals reported as having campylobacteriosis are hospitalised and sequelae include not only severe stomach cramps and diarrhoea but in up to two-thirds of cases musculoskeletal, joint swelling or sensory problems [10]. In the UK it has been reported that *Campylobacter* contributes to 15% of all Guillain-Barré Syndrome cases [11] and >80 deaths annually [12]. This all causes considerable demands on health services, economic costs and impacts on those infected and their families and carers.

Human *Campylobacter* infections may have been detected as far back as 1880 in diarrhoeal infants in Germany [13]. However, it was not until 1977 when Skirrow developed a routine plating method that clinical microbiological labs were readily able to isolate *Campylobacter* from stool samples [14]. In the UK *Campylobacter* reports increased linearly during the 1980's [15] and this continued during the 1990's until a peak was reached in 2000, then followed a decline to 2004/5 followed by a steady increase to the current day [5]. However, this trend in human infection is considerably complex with large increases in disease incidence in the elderly reported over the last 20 years, whilst incidence in young children has actually fallen [6], [16]. It is currently unclear which of the factors of emergence mentioned above are driving this disease pattern.

*Campylobacter* is zoonotic and is found in a very wide range of sources including farm and wild animals, birds and pets [13]. Cases are predominantly sporadic with few outbreaks and secondary transmission in humans is rare [17]. The aetiology is complex and identification of risk factors has traditionally been by empirical and analytical epidemiology predominantly utilising case control methodologies [18]. These risk factors denote the statistical likelihood of being ill (or not ill) [19] and can either relate to the “source” (e.g food vehicle or host reservoir) or the “human population” (e.g. age, gender, location etc.) [20]. However, a risk factor approach has a number of weaknesses [21]. First, these risk factors are usually more specific to the particular infectious disease than the general factors for emergence listed above. Second, case-control studies are usually conducted at a single time-point (or over a relatively short period) and they do not provide information on emergence of infectious disease over time. Third, the analyses assume that populations are simple collections of individuals that are independent (i.e. do not interact) [22].

A meta analysis [23] of case control studies on *Campylobacter* from across the world found that international travel, followed by consumption of undercooked chicken, environmental exposure (drinking water, recreational water, contact with bird droppings) and direct contact with farm animals (particularly associated with young children) and pets were significant risk factors. Other important factors included pre-existing chronic disease, eating chicken in a restaurant, eating poultry and consuming unpasteurized dairy products.

A case-control study in North-East Scotland [24] reported that proton pump inhibitors (PPIs) (Odds ratio (OR) 2.4), overnight stay outside study area (OR 2.03), contact with farm animals (OR 1.50), pets at home (OR 1.23), private water supply (OR 2.98), barbeque and picnic (OR 1.47) and diving in the sea (OR 4.14) were associated with disease whilst consumption of pre-packed ready to eat foods was protective (0.60). Unfortunately, additional risk factors such as preparing chicken from raw, consuming undercooked chicken etc. were not collected in this study. There is the potential to compare risk factor data (e.g. originating from case control studies) over different time points. This could be done in a case-case format with one time point being the reference. This would enable identification of changes in risk factors over time but this has yet to be attempted for human campylobacteriosis.

More recently with the advent of DNA sequence based typing methods such as multi-locus sequence typing (MLST) it has been possible to attribute human cases to source (either at the point of reservoir or retail food level). MLST studies from north-west England [25], Scotland [26] and New Zealand [27] have all identified chicken as the main reservoir source of human disease (50% to 80%) with the most common strains found in humans also being the most common in retail chicken. These studies also identified ruminants as an important reservoir source and further epidemiological work demonstrated that this was particularly the case for young children in rural areas [28] especially during the spring peak of infection [29]. Source attribution (employing microbial sub-typing) is again generally based on a single time point (or short period) though dynamic temporal attribution is now being reported as data become available [30].

It is now possible to integrate analytical epidemiological methods and microbial sub-typing. For example Gras and colleagues [31] combined risk factors from a case-control study with MLST based source attribution data. They found that consuming chicken was a risk factor for campylobacteriosis caused by chicken associated genotypes. Also, that ruminant associated genotypes were associated with occupational exposure to animals, barbecueing in non-urban areas, consumption of tripe and never/seldom consumption of chicken.

Another approach is to use the source attribution data in a case-case format where for example there can be comparison between chicken and non-chicken genotypes. This has been carried out for *C. jejuni* [32] where cases attributed to poultry sources were associated with adults, females, high population density areas, the winter and foreign travel. For *C. coli* [33] cases associated with poultry were also associated with being female. But again all of these studies are considering the aetiology of human campylobacteriosis at a single timepoint or over a short period.

To understand the pattern of disease emergence it is first necessary to obtain the temporal dynamics of the factors that drive this emergence [1]. Table 1 uses Morse's emerging factors as a framework on which to append specific examples for human campylobacteriosis. To these, explanatory variables can be assigned which include both risk factors from case control (or case-case) studies and time series data. The aim of this paper is to use *Campylobacter* as an exemplar infectious disease that has emerged over the last 30 years to both evaluate the utility of this framework as well as evaluating its explanatory power in unravelling the mechanisms of disease emergence/re-emergence. The study will focus on Scotland, particularly the north-east (i.e. the Grampian region) where data sets are available over extended time periods and where there is a considerable burden of disease.

**Table 1:** Factors and explanatory variables for infectious disease emergence exemplified for human campylobacteriosis.

Emergence Factor	Specific example for *Campylobacter*	Explanatory variables	
		Time Series	Risk Factor (Case control or Case-Case)
Ecological changes (including those due to economic development and agricultural land use)	Intensification of on farm chicken production	No. of chicks being placed into production*	
Human demographics, behaviour	Ageing population	Census statistics Campylobacteriosis rate by age*	Age*
	Rural/Urban population	Census Statistics Campylobacteriosis rate by rurality*	Rurality*
	Deprivation	Census statistics Campylobacteriosis rate by deprivation index	Deprivation*
	Chronic diseases	Population use of PPI's* Hospitalisation*	Use of PPI's*
	Eating/drinking habits	Consumption of chicken (household)*	Consumption of undercooked chicken, Eating chicken (at a restaurant) Consumption of unpasteurized dairy products BBQ & Picnic Drinking from PWS
	Interaction with domestic animals, pets and wildlife		Contact with farm animals Contact with pets Contact with birds (droppings)
	Recreation		Recreational water, diving in sea
International travel and commerce	Contracting disease whilst on travels	International travel survey* Domestic travel survey	Overnight stay outside home International travel* National travel (involving overnight stay outside of home)
Technology and industry	Modern food production and processing	Prevalence/load on chicken after slaughter and retail*	
Microbial adaptation and change	Change of genotypes in humans and animals	Dynamic source attribution*, Genetic distance*, Genetic diversity*, Phylogenetic evolution*	
	Antibiotic resistance	Surveillance over time	
Breakdown in public-health measures	Commercial Restaurant	Outbreaks e.g. associated with chicken liver pâté	

* Variables used in the current study.

## Materials and Methods

### Data

#### Clinical cases

In Scotland *Campylobacter* confirmed laboratory reports are notifiable (Health Protection Scotland. Available: www.hps.scot.nhs.uk/publichealthact/Index.aspx. Accessed 2013 Oct 2.) and the weekly reported cases of human campylobacteriosis between the years 1990 and 2011 (n = 112,230) were obtained from Health Protection Scotland (HPS). The collection of these data is part of a national surveillance system, where diagnostic laboratories throughout Scotland provide HPS with weekly reports of detected infections. These data are stratified by regional health board (n = 15 pre-2006, 14 afterwards) and into groups (young children (0–4), children (5–14), adults (15–64) and the elderly (65+). Subsets of human case reports (Grampian 1997–2010 totalling 10,247 cases and Scotland 2000–2006 totalling 34,947 cases) also included details of postal sector, gender and reporting date. Isolates were further obtained from the following subsets of human cases and genotyped by MLST (Grampian, 2001, n = 172; Grampian 2005–2007, n = 1452 and Grampian 2010–2012, n = 1292). Clinical case data were combined with human population data (see below) to determine disease incidence.

#### Human population

Middle year population estimates (1991–2011) at health board level stratified by 5 year age groups and gender were obtained from the General Register Office for Scotland (GROS. Available: www.gro-scotland.gov.uk. Accessed 2013 Oct 2.). In addition these data were acquired at postcode sector level from the 2000 census.

#### Hospitalisation

The number of hospital episodes in Scottish residents with a diagnosis of *Campylobacter* enteritis (ICD-10 code A045) by calendar year (1997–2012, n = 7291) and stratified by age was obtained from the Information Services Division (ISD), National Health Services of Scotland, Edinburgh, UK.

#### Poultry industry and household purchases of chicken

The number of chicks placed on commercial broiler farms in the UK was obtained from DEFRA during 1993–2012 (GOV.UK. Available: www.gov.uk/government/publications/poultry-and-poultry-meat-statistics. Accessed 2013 Oct 2.). The average amount (kg) of poultry purchased by each household per year from 1988 to 2012 was obtained from DEFRA's Family Food Survey (National Statistics. Available: https://statistics.defra.gov.uk/esg/publications/efs/datasets/default.asp. Accessed 2013 Oct 2.).

#### Integration of case control data, foreign travel and PPI time series datasets

Case-control data from north-east Scotland gathered between 2005–2007 [24] were used to infer the incidence of human campylobacteriosis in Scotland associated with foreign travel and PPI risk factors (these risk factors were selected because they had OR >1.5 and >15% of cases were exposed). Data on foreign travel visits (1990–2011 obtained from the international passenger survey, Transport Scotland. Available: http://www.transportscotland.gov.uk/. Accessed 2013 Oct 2.) and PPI dispensing rates (1993–2011 obtained from ISD, Edinburgh, UK) were used to infer the incidence of human disease associated with these risk factors during the study period.

### Microbiology and Genotyping

#### Sampling retail chicken and animal hosts

Retail chicken meat was sampled in 2001, 2005–07 and 2010–12 as described previously [34]. Samples were collected from rural and urban areas across north-east (Grampian) and also south west Scotland (2005–07 only). A full list is in Table S1. In all cases fresh faceal mammalian (25 g) and avian (<5 g) grab samples from the field and abattoir were collected. Samples were transported chilled (4°C) to the laboratory for immediate analysis [35].

#### Microbial isolation

The presence or absence of *Campylobacter* was determined by homogenising faecal aliquots (10∶90 w∶v) in *Campylobacter* enrichment broth (see below) microaerobically in an atmosphere of 10% CO_2_, 5% O_2_, balance N_2_. Enrichment broth comprised 100 mL volumes of nutrient broth base (Mast, Bootle, United Kingdom) with 5% horse blood, growth supplement (Mast Selectavial SV61), amphotericin (2 mg/mL), cefoperazone (15 mg/mL), and trimethoprim (10 mg/mL); polymixin B (2500 IU/L) and rifampicin (5 mg/mL) were added, and the broths incubated for 2 days at 37°C (Bull et al., 2006). All antimicrobials were purchased from Sigma-Aldrich (United Kingdom). Enrichment broths (0.1 mL) were plated onto modified *Campylobacter* Blood-Free Selective Agar Base (CCDA base, CM0739; Oxoid, United Kingdom) with CCDA selective supplement (SR 155; Oxoid) incubated microaerobically for 2 days at 37°C (minimum detection level was therefore 0.1 cfu/g).

#### Genus and species confirmation

Genus confirmation of *Campylobacter* spp. was achieved by Microscreen latex (Microgen, Camberley, United Kingdom) agglutination test. Individual colonies were stored (−80°C, nutrient broth, glycerol added to 15% [v/v]). Bacterial DNA from latex test-positive isolates was prepared using a DNA release method (Chelex resin; catalogue no. 142-1253; Bio-Rad, United States). *Campylobacter* species was confirmed using the isolates ST as determined by MLST.

#### MLST

Genotyping by 7-locus MLST was performed as described previously [26]. Allele profiles and sequence types (ST's) were assigned using the *Campylobacter* MLST profile database (PubMLST. Available: www.pubmlst.org. Accessed 2013 Oct 2.).

#### Clinical isolates from north-east Scotland

Isolates were received on CCDA plates from Aberdeen Royal Infirmary. Single colonies were plated on modified CCDA and incubated, genus and species confirmed and typing by MLST were all carried out as described above (See Table S1).

### Epidemiological Data Analysis

#### Demographic time series analysis of *Campylobacter* cases

This was performed at postcode sector (2000–2006, n_pcs_ = 937) and health board level (1990–2006, n_hb_ = 12 and 2007–2011, n_hb_ = 11). At postcode sector level urban cases were defined as those with >200 persons/km^2^, otherwise defined as rural. The incidence for both the rural and urban cases was calculated across Scotland for each study year. From this the urban/rural ratio was calculated along with the 95% binomial confidence intervals. At health board level the total urban and rural populations were determined using GROS data in ArcGIS 10.0 (ESRI, Redlands, California, USA). The *Campylobacter* cases were designated as urban or rural in proportion to the urban/rural population in each health board. All urban designated cases were summed across Scotland and divided by the urban population to obtain the urban incidence. This was repeated for the rural cases. Finally the urban/rural ratio and confidence intervals were calculated as above and this was repeated for each year. This was also performed by age group (0–4, 5–14, 15–64, 65+, see Table S2).

#### Case-case analysis

Two analyses were performed on reported cases from the Grampian Region of Scotland. These compared the lower incidence period (2002–2006, 3313 cases) and the resurgence period (2007–2010, 2885 cases) with the peak period (1997–2001, 4049 cases). Five parameters were available as putative risk factors; (1) age (0–4, 5–14, 15–64, 65+), (2) gender (male or female), (3) season (summer - June to August - or the rest of the year), (4) rural or urban human population density (rural ≤200 individuals/km^2^ and urban >200 individuals/km^2^) and (5) deprivation - deprived (Carstairs index >−3) or affluent (Carstairs index ≤−3) – see Table S3. Analyses were carried out using univariate and multivariate logistic regression employing the epidemiological modelling software package EGRET (version 2.0.3, Cytel Software Corporation, Cambridge, MA, USA). Results for each risk factor were considered as statistically significant when P<0.05. Factors from the univariate analyses with a P value of <0.25 were used in the multivariate analysis.

### Molecular epidemiology

#### Nei's genetic distance

Standardized genetic distances (d_1_) between clinical and host sources for the periods 2001, 2005–07 and 2010–12 were determined using the method of Nei [36] and applied to genetic locus data by the method of Manly [37]. The level of significance was obtained using randomization tests (10,000 iterations). Briefly, the data from the populations (e.g. clinicals and chicken) were randomized in Excel using PopTools (PopTools. Available: http://www.cse.csiro.au/poptools/. Accessed 2013 Oct 2.) and the genetic distance calculated. This process was then repeated 10,000 times using the Monte Carlo Excel add-in @RISK (Palisade Ivybridge, United Kingdom). The posterior distribution of genetic distances was then compared with the non- randomised genetic distance to obtain the level of significance (P value).

#### Source attribution

The probable reservoir origin of *Campylobacter* was investigated using MLST sequence types (ST's) investigated with structure genetic population software [38]. Using this method, isolates can be probabilistically assigned to ancestral populations based on ST frequencies. Source datasets of *Campylobacter* strains with known origins were used as a reference population and clinical isolates were attributed to this based on ST similarities (see Table S1). This source dataset comprised data collected in three time periods 2001 (chicken (n = 84)), 2005–07 (chicken (n = 277), cattle (n = 104), sheep (n = 97), wild birds (n = 188) and pigs (n = 40)) and 2010–12 (chicken (n = 354), cattle (n = 158), sheep (n = 196)) obtained from the CaMPS study and it's extension i-CaMPS [39].

#### Genetic diversity

Genetic diversity was determined by Simpson's index [40] where a value of 0 indicates homogenous ST's and a value of 1 indicates a heterogeneous population with maximum diversity. Confidence intervals were calculated using a bootstrap method from the PopTools add-in for Microsoft Excel (PopTools. Available: http://www.cse.csiro.au/poptools/. Accessed 2013 Oct 2.).

#### Geneaolgy of isolates

The clonal genealogy of *Campylobacter* sequence types (ST's) was estimated using the model-based approach for determining bacterial microevolution employed in clonalframe, version 1.0 (Xavier Didelot. Available: http://www.xavierdidelot.xtreemhost.com/clonalframe.htm. Accessed 2013 Oct 2.) [41]. This approach incorporates both point mutation and horizontal gene transfer events. The program was run with 50,000 “burn-in” iterations which are discarded to minimise the effects of initial values followed by 50,000 data collection iterations. The consensus trees represent combined data from three independent runs, with 75% consensus required for inference of relatedness. Two trees were generated, one for *C. jejuni* and the other for *C. coli* [42]. Due to the large datasets singleton strains were removed (370 and 62 for *C. jejuni* and *C. coli* respectively).

## Results and Discussion

**Figure 1:**
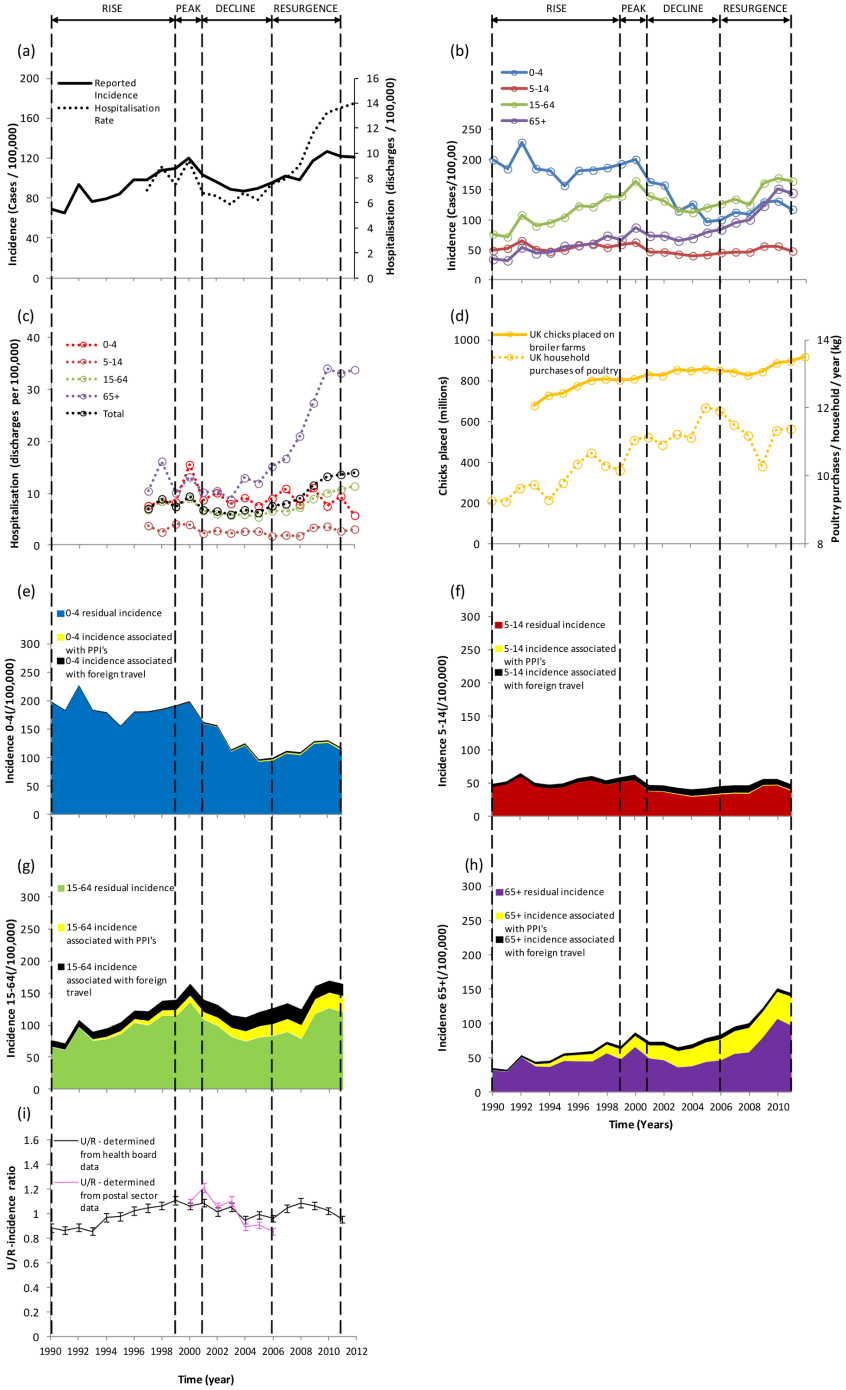
Time series data (a) incidence and hospital discharge rates of human campylobacteriosis in Scotland, (b) incidence stratified by age, (c) hospitalisation rates stratified by age, (d) number of chicks placed into UK broiler farms and poultry purchases by household, (e)–(h) age stratified incidence associated with foreign travel, use of proton pump inhibitors and the residual (not explained by the former two factors) and (i) urban/rural ratio of incidence aggregated from Health Boards (1990–2011) and postal sectors (2000–2006) with 95% binomial confidence intervals.

This comprises four sections. The first uses the explanatory variables obtained from Morse's emergence factors to give an informed explanation of the different phases of the human campylobacteriosis epidemic in Scotland. This involved four phases (epidemic rise (1990–1999), peak (2000–01), fall (2001–06) and resurgence (2007–11) during which there was a >300% increase in disease incidence in the elderly and a 50% reduction in young children (Figs. 1(a) and (b)). The second section describes the clonalframe results. The third gives an international perspective and identifies limitations of the study. The fourth considers future trends and the general utility of Morse's factors for disease emergence.

### Explanation of the phases of emergence

#### Epidemic rise of human campylobacteriosis (1990–1999)

During this period the number of human cases increased by 75% (Fig. 1(a)), hospitalisation rates were relatively high, the poultry industry increased in size (Fig. 1(d)) by >18% and availability of poultry meat provided a relatively inexpensive, nutritious and healthy source of protein that the public readily embraced [43]. This can be seen by the 10% increase of household purchases during this period (Fig. 1(d)). The increase in human cases took place across the country but was greatest in urban areas (Fig. 1(i)). This latter fact is likely to be more consistent with an increasing foodborne (e.g. chicken) source of infection. It has already been established that rural cases of disease are more likely to be associated with non-chicken (e.g. ruminant or wild bird) genotypes [29]. This is especially the case for young children (<5 years) who are in effect sentinels of environmental infection pressure. This is further emphasised by the static level of cases in young children (Fig. 1(b)) during this period which provides further evidence to show that the epidemic rise cannot be explained by environmental sources and that a foodborne route (e.g. chicken) is more probable.

The number of visits abroad increased by 60% during this period. Assuming the same fraction of these resulted in acquiring *Campylobacter* infections as reported in the case-control study [24], 9% of human cases are likely to be associated with travel abroad throughout the 1990's. PPI's were first introduced in 1989 and usage increased during the 1990's [44]. By 1999 an estimated 6% of cases were associated with PPIs and this includes 22% in the elderly. It is recognised that gastric acidity is an important defense mechanism against ingested pathogens, *Campylobacter* is sensitive to acid stress [45] and PPI's have also been associated with an increase of other gastrointestinal infections including *C. difficile* [46]. Ideally case-control information, molecular genotyping of human cases and animal populations carried out during this period would provide further evidence to identify and explain changes in the putative sources of human campylobacteriosis.

A final explanation for the increase of *Campylobacter* cases during this period could be due to enhanced awareness in public health or a change in methods (e.g. at clinical diagnostic labs). However, this seems unlikely for two reasons. The first, that the methodologies in public health/microbiology labs did not change over this period and the second that the ratio of community to reported *Campylobacter* cases were not significantly different in the intestinal infectious disease studies carried out between 1993–96 and 2008–09 [9], [47].

#### Peak period 2000–01

**Figure 2:**
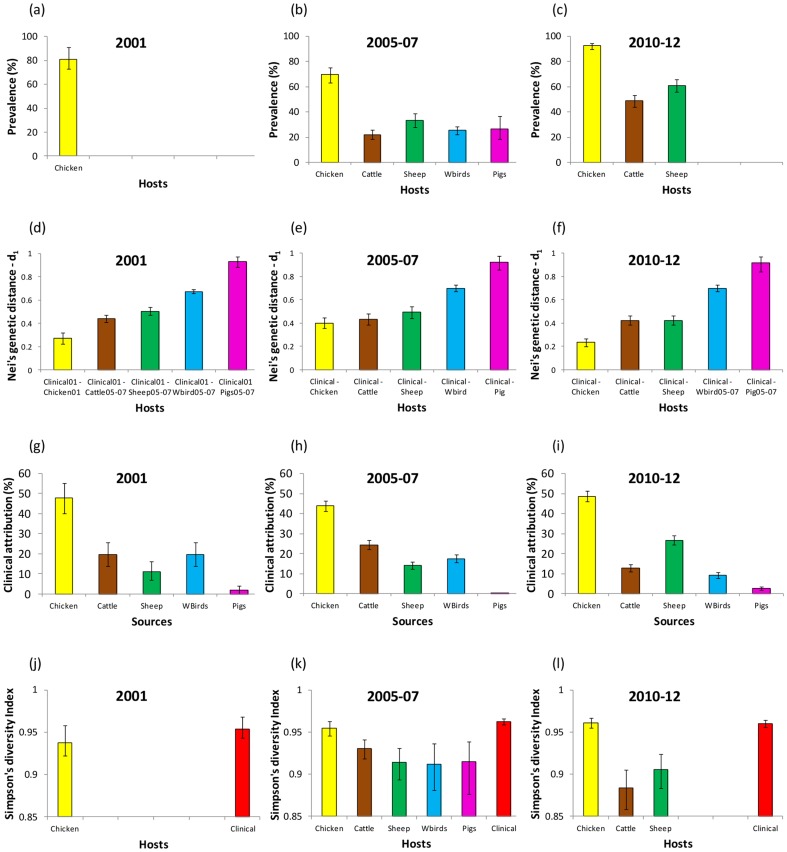
Host prevalence (a)–(c), genetic distance between human clinical and host genotypes (d)–(f), source attribution by structure (g)–(i) and Simpson's index of diversity (j)–(l) for the three time periods (2001, 2005–07 and 2010–12).

**Figure 3:**
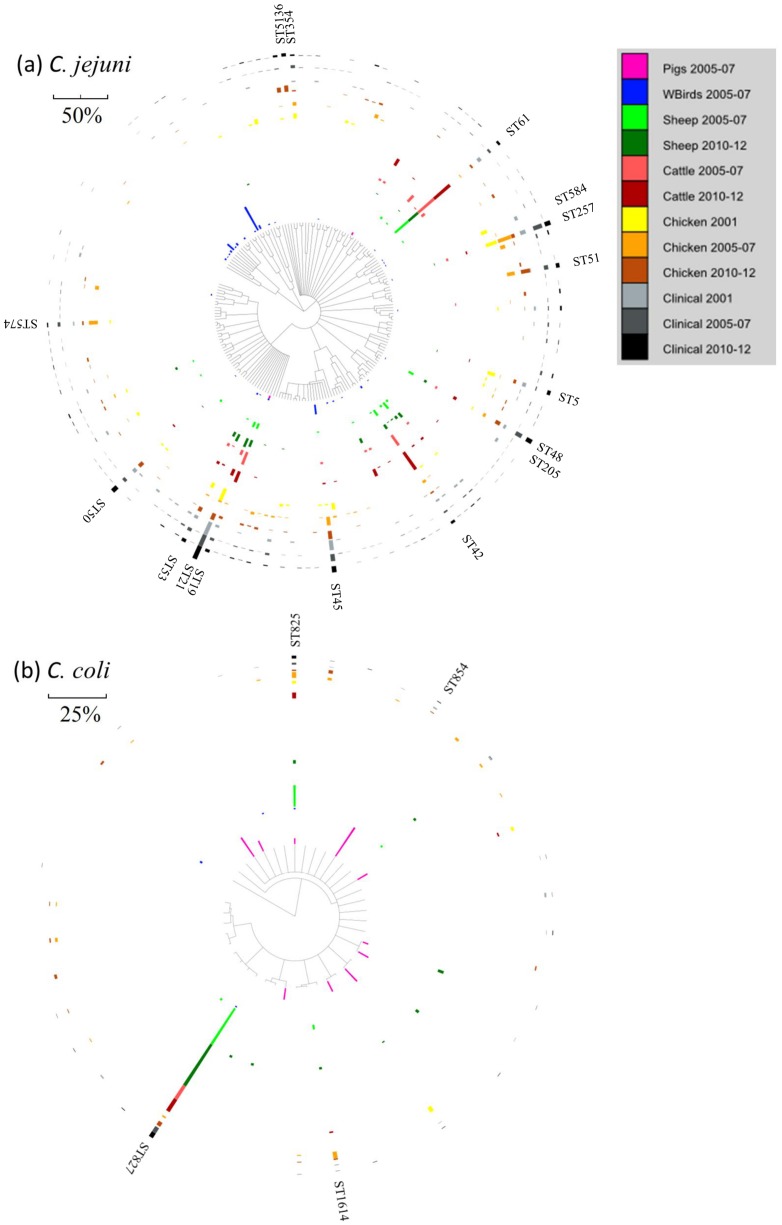
clonalframe geneaologies for (a) *C. jejuni* and (b) *C. coli* for the three study periods. The abundance (%) of each genotype in each host, for a particular time period, for combined *C. jejuni* and *C. coli*, is denoted by length of scale bar. Singleton ST's were removed from the analysis.

Human incidence peaks (Fig. 1(a)), prevalence in chicken was high (81%) and both genetic distance and source attribution indicate that retail poultry is the most important source of human campylobacteriosis (Fig. 2). Further, Simpson's diversity index was indistinguishable between clinicals and chicken (P>0.05). In addition, five out of the top ten clinical and chicken ST's were the same (Fig. 3 and Table S1). ST584 is present in both humans (2.9%) and chicken (6%) but disappears in subsequent years. The urban/rural incidence is also high (>1.0) during this period suggesting that a food-borne rather than environmental source is responsible.

#### Decline and trough 2001–06 (approx)

There was a drop in human incidence across all age groups (particularly striking in the <5 year olds where there was a 35% fall) except the elderly (5% increase). Hospitalisation rates were static during this period (Fig. 1(a)). Molecular attribution indicates that chicken is still the most important source of human disease but genetic distance indicates that cattle and chicken are both important, whereas wild birds and pigs are relatively unimportant. The dramatic fall of 35% in young children comprises a reduction of 5% and 53% in rural and urban disease incidence respectively. It has already been established [28] that environmental sources are important for cases in young rural children and hence it seems likely that non-foodborne routes of infection are still prominent throughout this period. The case-case temporal study shows that for the overall population the urban/rural ratio decreases in this period compared with 1997–01 (Table 2, see also Fig. 1(i)). This is due to the relatively high reduction in urban compared to rural incidence suggesting that there could be a drop in cases associated with chicken. This concurs with a significant increase (P<0.05) in Nei's genetic distance when comparing clinical and chicken isolates between 2001 and 2005–07 and a decrease (although not significant, P>0.05) in clinical isolates attributed to chicken (Fig. 2.).The increase in the elderly may be explained by the 50% increase in dispensing of PPI's as well as omeprazole (Prilosec) becoming available over the counter in 2004 [48]. Despite human incidence decreasing, travel associated cases increased by an estimated 5%. Also, household purchases of poultry are still strong and the prevalence in chicken, though lower than in 2001, is not significantly so (P>0.05). There is a change in the distribution of genotypes over this period [34] (see Table S1) which may play a role in explaining the changes in human disease: genotypes may have different dose responses [49] and different capacities to cause human morbidity [50].

**Table 2:** Results of the logistic regression for the case-case studies.

		Comparing 2002–06 (cases) versus 1997–01(Ref. period)			Comparing 2007–10 (cases) versus 1997–01 (Ref. period)		
Factors	Group	O.R.	C.I. (95%)	P-value	O.R.	C.I. (95%)	P-value
(A)Univariate							
age^a^	young	1^b^	-	-	1^b^	-	-
	old	1.112	1.042–1.186	0.0013* †	1.380	1.288–1.478	<0.0001* †
gender	male	1^b^	-	-	1^b^	-	-
	female	0.931	0.49–1.020	0.1248†	0.960	0.873–1.057	0.4083
season	rest of year	1^b^	-	-	1^b^	-	-
	summer	0.972	0.879–1.075	0.5799	0.885	0.796–0.984	0.0234* †
location	rural	1^b^	-	-	1^b^	-	-
	urban	0.895	0.815–0.982	0.0196* †	0.937	0.850–1.033	0.1913
Carstairs	affluent	1^b^	-	-	1^b^	-	-
	deprived	0.914	0.833–1.002	0.056†	0.929	0.845–1.024	0.1377
(B) Multivariate							
age^a^	young	1^b^	-	-	1^b^	-	-
	old	1.215	1.051–1.197	0.0006*	1.380	1.285–1.476	<0.0001*
season	rest of year				1^b^		
	summer				0.898	0.807–1.000	0.0491*
location	rural	1^b^	-	-			
	urban	0.880	0.801–0.967	0.0077*			

(A) Odds ratios and their associated P-value for all the selected cases in the univariate models.

* Factors with P<0.05 are considered as significant.

† Factors with a P<0.25 are entered in the multivariate model.

(B) Odds ratios and P-values for the final multivariate models. Previous steps, consisting in removing one by one the factors with the highest P-value at each step, are not shown. The program used to execute the analysis gave P = 0.002 for the overall model fit comparing 2002–06 with 1997–01 and P = <0.0001 comparing 2007–10 with 1997–01.

a Humans are grouped into four age groups with the reference group being young children (0–4 years) (see Table S2) and the odds ratio indicates the relative amount by which the odds of the outcome changes when the value of the predictor value is increased by 1.0 unit.

b Reference group.

#### Resurgence 2007–2011

There was a 20% increase in human incidence which can be seen across all age groups and particularly the elderly (50% increase) who also present a dramatic 100% increase in hospitalisations. The case-case study (Table 2) also showed an increase in elderly cases compared with 1997–01 (OR = 1.38, P<0.0001). PPI dispensing increased by 30% (numbers of over the counter sales probably increased as well though data are unavailable) and it is likely that >30% of cases in the elderly were associated with PPI's (Fig. 1(h)). Further, at least one quarter of the increase in incidence during this period was likely to be associated with PPIs (Fig. 1(h)). Although houshold purchases of poultry decreased by 5% (Fig. 1(d)) the prevalence of *Campylobacter* in chicken was highest (93%, P<0.01) (Fig. 2(c)). Cattle and sheep also have high prevalence compared with the previous period (P<0.0001), but source attribution and genetic distance identify chicken as the most important putative source for human disease. Further, since 2010 *Campylobacter* has become the most frequent agent of foodborne outbreaks in England and Wales [51]. The majority of these outbreaks are associated with chicken liver pâté or parfait and so the preference of lightly cooking could well be contributing in part to the rise of cases seen in the human population. ST5136 appears to be a new strain (only found in this period Fig. 3(a)) and is associated with chickens and humans.

### clonalframe derived geneology

Clinical isolates are widely distributed across the *C. jejuni* phylogenetic tree (Fig. 3(a)). To a lesser extent this is the case for chicken and to an even lesser extent this is the case for cattle and sheep. Some ST's can be considered generalists and are found in most hosts and time periods (e.g. ST21, ST53, ST48, also ST19, ST61, ST42 (these are more common in cattle and sheep), also ST257 (more common in chicken) and ST45 (more common in chicken and birds)). Further, some ST's appear to be found only in clinical and chicken isolates (ST51, ST354, ST574, ST5136). Pig *C. jejuni* ST's are rare and there is a cluster of wild bird sequence types (see Fig. 3(a)) that are relatively rare in the other hosts. Similar percentages of clinical 16% (29 present in all three periods/180 present in at least one period) and chicken 14% (13/93) non-singleton ST's are present throughout the 3 time periods of the study (P = 0.725). Cattle and sheep isolates were genotyped during only two periods of the study (2005–07 and 2010–12) but it was found that for non-singletons 40% (12/30) cattle and 43% (16/37) sheep ST's were present in both time periods.

Clinical, chicken, sheep and pig isolates are found across the *C. coli* tree (Fig. 3(b)) whereas there are infrequent wild bird (n = 4 and 8 singletons) and cattle (n = 4 and 2 singletons) ST's. Some genotypes (e.g. ST825 (all), ST827 (all except pigs), ST1614 (all except pigs and birds)) are found in most hosts for at least two of the time periods. ST854 is common (15%) in pigs but rarely found in clinicals and chicken. A number of ST's (n = 5) are only found in pigs.

### International comparisons and study limitations

This analysis of the *Campylobacter* epidemic is likely to be representative of the UK as a whole where there have been similar changes in incidence [6]. Evidence exists from other countries on changes in *Campylobacter* incidence rates over the last 25 years. For example in New Zealand campylobacteriosis increased to a rate of >350 per 100,000 prior to 2006 which was contemporaneous with rising poultry consumption [30]. Human incidence fell by 50% post-2006 coincident with a range of voluntary and regulatory poultry interventions. This resulted in a 74% reduction in cases attributed to poultry with a higher proportion of the remaining cases being rural and associated with ruminants. In Iceland [52] before 1996 all retail poultry was frozen as a control measure for salmonellae, which coincidentally reduced *Campylobacter* loading resulting in 10 cases per 100,000 of the population reported annually. In 1996, fresh poultry was allowed to be sold and by 1999 the campylobacteriosis rate had risen to 120 per 100,000. Subsequently, flocks were tested before slaughter and for those that were found to be positive the birds were frozen prior to retail. This intervention, together with on-farm measures, reduced the incidence of human campylobacteriosis close to that found before 1996 [53]. In the USA, campylobacteriosis has fallen by 27% since 1996 [8]. Most of this change was between 1996–2001, and this is thought to be primarily due to improved sanitation in broiler processing, particularly the addition of chlorine in the spin chiller, an intervention that was also used in New Zealand.

Elucidating the past aetiology of epidemics is by necessity an incomplete science since it is attempting to infer history from partial datasets utilising methods from a number of scientific disciplines (e.g. microbiology, epidemiology, molecular biology etc) that are still under development. For example the case-control study in NE Scotland [24] did not investigate specific risk factors for chicken (e.g. undercooking or eating chicken at a restaurant) which would have provided valuable information to the current study. However, data on consumption of chicken together with genotyping data (e.g. source attribution and Nei's genetic distance) provided information on exposure at a population level and likely reservoir source of disease. Other risk factors which were not possible to include in the temporal analysis across the study included drinking unpasteurised milk, recreational water exposure and drinking water from a private water supply. The sale of unpasteurised milk and cream has been banned in Scotland since 1983 and so there are unlikely to be many cases of campylobacteriosis via this vehicle. The only recreational water exposure risk factor of statistical significance from the NE Scotland case-control study was diving in the sea. However, this comprised only a few individuals and although this may be an important risk factor for this group it is likely to have little impact on the total number of cases of campylobacteriosis. Consumption of water from private water supplies was found to be of significance in the NE case-control study conducted during 2005–07. It is unknown how disease associated with this risk factor may have changed over time but it is known that there have been a number of initiatives and grants to improve private water supplies in Scotland (e.g. treatment by UV light).

It was possible to combine the PPI and foreign travel case-control data from NE Scotland with national time series data for PPI dispensing rates and foreign travel visits respectively to infer the associated disease burden throughout the study period. This assumed that there would be no confounding when scaling from region to nation. It has been previously shown that there is no significant difference between the distribution of clinical *Campylobacter* genotypes between regions and nationally [39]. However, national case control data on the risk factors would validate the results presented here and provide information at the level of the individual which would complement our inferences made at the level of the population.

There is also the potential for reporting bias which can suggest that there are changes in disease incidence (e.g. over time, between age groups, between urban and rural populations etc) when in reality there are none. However, post 1990 in Scotland there have been no changes in public health policy and clinical lab diagnostics. Also, similar changes in overall disease incidence, as well as disease incidence stratified by age, have been reported for England and Wales [16].

It is clear that collections of genotyped human and animal isolates combined with risk factors from case-control (or/and case-case) studies and time series data sets are required across the period under study. This of course requires signficant financial resources which must be set against competing requirements (from other infectious diseases and elsewhere) from public health, food safety and other governmental agencies. However, a coordinated approach is required to hasten understanding of a pathogen such as *Campylobacter* which is an issue across the world and this is currently lacking.

### Predicting the future and determining the utility of Morse's factors for infectious disease emergence

This paper has provided explanations of what has happened in the past. Of greater importance is to apply this knowledge to predict what may happen in the future and to act on this by developing strategies to reduce the burden of disease. It is therefore important to understand how Morse's factors for disease emergence may change. In terms of ecological changes due to economic/agricultural developments it is unlikely that there will be further intensification in poultry production in the developed world and only modest increases are forecast for the UK and USA over the next ten years [54]. There seems to be little scope for controlling *Campylobacter* in the wider agricultural environment because of its many domestic and wildlife reservoirs.

Human demographics will change in the developed world with an increasingly elderly population. For example in Scotland between 2010 and 2020 it is estimated that there will be 15% and 23% increases in the 60–74 and 75+ age groups respectively. This could lead to both a 7% and 5% increase in hospitalisations and reported cases respectively (assuming everything else is held constant). PPI dispensing still appears to be increasing and this combined with the growing numbers of elderly will likely result in further increases in cases and hospitalisations. However, a recent study [55] indicates that PPIs are a marker for human campylobacteriosis and other factors must be responsible for the increased risk in this cohort. There is the potential for human behaviour to change. For example attitudes to *Campylobacter* from regulatory and industry are changing. There is a recognition by industry that *Campylobacter* contamination is a problem that needs to be dealt with (British Poultry Council. Available: www.britishpoultry.org.uk/?p=1857. Accessed 2013 Oct 2.). In the UK, the EU and elsewhere government led strategies are being developed and research carried out to try to reduce the prevalence and loading of *Campylobacter* in chicken [56]. It is not yet possible to assess how successful these will be.

International travel has reduced during the recent recession (2008–9 and 2011–12). It is of course very difficult to predict economic recovery but when this does take place then international travel will increase and it is likely that human campylobacteriosis will increase as a result. Technology and industry developments include development of poultry breeds that are resistant to *Campylobacter* or a vaccine [57]. However, these solutions appear to be at least 5–10 years away.

There is the likelihood of microbial adaption and change in the population of *Campylobacter*. This has already been seen in changing genotypes and the appearance and disapperance of particular ST's found in humans and poultry. There is also concern of increasing antimicrobial resistances in human isolates (e.g. 30% increase in ciprofloxacin resistance between 1995 and 2008 largely caused by veterinary application in agriculture [58]). Improved public health measures (e.g. improving kitchen hygiene in both commercial and domestic premises and proper cooking of chicken liver pâté) can be readily attempted through education. However, again it will be difficult to gauge the potential success of these.

All of the six emergence factors considered (Table 1) play a role in the changing *Campylobacter* epidemic, demonstrating the multifactorial nature of this infectious disease. The relative importance of these factors is confounded by interactions between them (e.g. ecological change through intensification of chicken production has offered greater potential for *Campylobacter* to reach humans whose risk of infection is exacerbated if they are being prescribed proton pump inhibitors). Currently there is no general theory for the emergence of an infectious disease that can encompass the myriad of factors (many of which are interacting) associated with this process. There is both germ theory and evolutionary theory which both have explanatory powers and contribute to understanding [59]. However, for *Campylobacter* even the utility of evolutionary theory is limited as far as humans are concerned because of the rarity of secondary transmission. There is the potential of using mathematical models to improve understanding of the epidemic. This would most likely require some combination of agent based (where the agents are the hosts) and spatially explicit contact network methods [60]. This has yet to be attempted but these types of models provide the opportunity to better understand the processes in disease transmission and the potential to simulate interventions.

In conclusion, Morse's emergence factors are demonstrated here to be of utility as a framework unravelling the aetiology of human campylobacteriosis [1]. It has been shown that these factors, made specific for *Campylobacter* (Table 1), can be both elucidated further and quantified through a series of explanatory variables (e.g. time series data and risk factors). Although, as admitted by Morse, the exact selection and wording of these emerging factors can be thought of as arbitrary and how they can be made operational is open to interpretation by the individual researcher, they do show promise across a gamut of emerging infectious diseases. Until a more parsimonious paradigm or framework can be established they are still a valuable tool in studying the emergence and re-emergence of infectious diseases.

## Supporting Information

Table S1 Molecular typing data and analysis (clinical, animal/food, attribution scores, genetic distance, common sequence types by host).
(XLSX)

Table S2 Urban-rural ratios of clinical cases stratified by age.
(XLSX)

Table S3 Case-case clinical data.
(XLSX)
